# Distribution Meeting of the Governors and General Council: Increase in Awards of £30,000

**Published:** 1916-12-23

**Authors:** 


### KING EDWARD'S HOSPITAL FUND FOR LONDON.

### Distribution Meeting of the Governors and General Council.

### INCREASE IN AWARDS OF £30,000.

A meeting of the Governors and General Council of King Edward's Hospital Fund for London for the purpose of awarding grants to the hospitals, convalescent homes and consumption sanatoria for the present year was held on Friday, the 15th inst., at St. James's Palace, the Viscount Iveagh, K.P., being in the chair.

Lord Revelstoke (the Honorary Treasurer) said that the net receipts of the year to December 11, apart from receipts on capital account, had amounted to £269,526 18s. 6d. This included £100,000 cash received from Sir Walter Trower in respect of the residue of the estate of the late Isabella Countess of Wilton, and the Fund had already had transferred to it securities amounting to over £22,000. As he indicated at the meeting in May last, the Finance Committee were retaining the cash uninvested, so as not to hamper in any way the discretion of the Council in deciding as to the use of the money in the event of any emergency.

Sir William Collins, in making the annual statement on behalf of the League of Mercy, said that so far as he was able to make an estimate, he thought there was no reason to doubt that the League would be in a position to make for the fourth year in succession a grant of £14,000 to the King Edward's Hospital Fund, and also to make a distribution of something like £3,000 in the Extra Metropolitan District, the total granted to the Fund in seventeen years being £244,000. He felt that in view of the splendid manner in which the hospitals had assisted the State by taking in thousands of wounded soldiers, the action taken by the League at the beginning of the war had been fully justified.

Sir William Church (the Chairman of the Distribution Committee) presented the report of that Committee, and Mr. Frederick M. Fry (Hon. Secretary) presented the list of awards (see pages 247-9).

Dr. Edwin Freshfield (the Chairman of the Convalescent Homes Committee) presented the report of that Committee, and Mr. John G. Griffiths (Hon. Secretary) presented the list of awards (see pages 249-50).

### The King's Congratulations.

Lord Iveagh, in moving the adoption of the report and awards, said:

My Lords and Gentlemen,—I have been asked by the Duke of Teck to say that his absence is due to work connected with the war, and by the Speaker to say that his special duties make it impossible for him to be here to-day.

First of all, I have the honour to read the following letter which has been addressed to the Governors by His Majesty the King, through Lord Stamfordham:—

"Buckingham Palace,
"December 12, 1916.

"I am commanded by the King, Patron of the Fund, to ask you to convey to the Council of King Edward's Hospital Fund at their meeting on the 15th instant his congratulations on the distribution of £170,000 this year. This, as His Majesty reminds you, is £30,000 more than last year's total, and £12,500 over the previous record achieved in the years just before the war.

"The King is much gratified at this evidence of the power of the Fund to give substantial assistance to the hospitals in a time of exceptional difficulty. He trusts that the public will help to maintain this record, and he hopes especially that there may be an increase in the ranks of annual subscribers, whose numbers are naturally depleted from various causes each year.

"His Majesty learned with much regret of the death of Mr. Algernon Sydney, who had done such good service to the Fund for many years; and he takes this opportunity of conveying to the Governors, Council, Officers, and all Committees of the Fund, as well as to the Visitors, his cordial appreciation of their work for the hospital service, now more important than ever to the welfare of his people.

"Yours very truly,
"Stamfordham.

"To the Presiding Governor,
"King Edward's Hospital Fund."

The Council will wish me to tender to His Majesty their humble thanks for his gracious message. We have all noticed with pride that His Majesty, the Patron of the Fund, has taken every opportunity of visiting the hospitals both abroad and in England, and has shown the deepest interest in their suffering patients and in the recent important progress of medical science. (Hear, hear.)

### A Record Distribution.

It is with great satisfaction that we find ourselves able to make the record distribution of £170,000. This is £30,000 more than last year, and £12,500 more than the previous maximum in the years immediately before the war. I am referring here to the ordinary distribution, apart from supplementary grants rendered possible by the generosity of Sir Ernest Cassel. As has been explained in previous years, the reduction in the total distribution in 1914 and 1915 was due, first to the decrease in the demand for building grants, and, second, to the danger of increasing largely the maintenance grants, before it was known which hospitals would be most affected by the financial difficulties arising out of the war. I say "danger," because there was also a third consideration—the question whether a larger total could have been maintained, or whether we might only have had to reduce our grants later on just when they were most needed. From that we have been saved, partly by the prudence, if I may venture to make that claim, of the Council, and partly by good fortune. We are able now to increase the distribution by £30,000, primarily through the receipt of legacies. Those of an ordinary character have this year amounted to £32,000, as against only £4,000 last year. But this, by itself, would not have been enough; and we certainly could not safely have distributed so large a proportion of these legacies of 1916 had it not been for the very munificent bequest by the late Isabella Countess of Wilton of the residue of her estate, received by the Fund during the year through Sir Walter Trower. The Council has, wisely I think, passed no resolution tying its hands as to the application of this legacy. No one can foresee what the difficulties of the hospitals may yet be as the result of the war, or what extra demands may be made on the Fund; and surely no one can accuse us either of reckless expenditure or of undue hoarding if in these

### KING EDWARD'S HOSPITAL FUND—(*continued*).

uncertain times the Finance Committee retains in cash such part of this legacy as was received in cash, and if the Council takes it into account in deciding how far the distribution can be increased without undue risk of violent fluctuations in the near future.

### A Steadying Influence.

For it is one of the functions of this Fund to serve as a steadying influence on the finances of the hospitals. I need hardly say that even £170,000 would not go far towards supporting the hospitals of London, if the contributions which they receive from their direct subscribers, as the result of their own efforts, were to show any very serious or permanent decline. The amount which the hospitals will get from the Fund in grants to maintenance, even in this record year, is only a little more than one-ninth of their total ordinary expenditure; while the very large increase we have been able to make in the maintenance grants will not cover the increase in their ordinary expenditure since the war began, reckoning only the year 1915. Therefore, I say, this Fund cannot maintain the hospitals: what it can do is to help to steady their financial position by providing an annual distribution, which may rise or fall with their needs, but is as far as possible free from involuntary fluctuations.

There is no doubt, moreover, that the need of the hospitals will continue to grow in the near future. The proportions I have given you relate to the expenditure of 1915, on which this year's distribution is necessarily based. The burden of 1916 will inevitably be greater; and if this Fund is to continue to do its part an increased distribution may become desirable. I commend this to the consideration of those, if any such there be, who, just because the operations of the Fund are so large, forget that the demands on it are proportionately great, and what is more, are, in the present circumstances, continually growing.

It is with much gratification that we learn of the success of the League of Mercy in collecting for the Fund the very large sum which Sir William Collins has mentioned.

We have also to express our indebtedness for the legacies of £20,000 received, under the will of the late Lady Brownlow, on the death of Sir Charles Brownlow; and £10,000 very kindly allocated to the Fund by the executors of the late Thomas Stephen Whitaker in the exercise of their discretionary power.

### Contributions from South Africa.

I should like also to make a special reference to a series of contributions, amounting in all to a total of over £270, which we have received at intervals throughout the year from the War Fund of the Citizens of East London, South Africa. This is a welcome recognition, coming from one of the distant parts of His Majesty's Dominions, of the great work which the hospitals of London are doing in aid of the sick and wounded sailors and soldiers.

The Governors are glad to see the reference in the Report of the Distribution Committee to the part which the hospitals are taking in the great fight against venereal diseases. This fund has, I believe, never singled out any particular section of the current expenditure of hospitals for special recognition in its grants to maintenance; which are made to the upkeep of each hospital taken as a whole. But the Committee recommend that the Fund, which by means of large capital grants was instrumental in bringing the Male Lock Hospital up to date, should now offer substantial assistance to the improvement of the Female Lock Hospital in Harrow Road.

### Working Men and their Sanatorium.

The Council will remember the special report made by the Convalescent Homes Committee in May last on the exceptional difficulties with which the Benenden Sanatorium was faced owing to the war. Fortunately the sanatorium was able to surmount those difficulties by its own efforts; but we are glad to be able to give it some special assistance in the reduction of its mortgage debt. This sanatorium, as you will remember, represents an effort on the part of the workers themselves to deal with the scourge of consumption, and as such it deserves our sympathy and support.

The inquiry into the question of pensions for hospital officers, which was begun by the Executive Committee before the war, was deferred for a time by the other preoccupations that press upon all of us. But it is progressing steadily under the expert chairmanship of Mr. W. J. H. Whittall.

The Fund has to regret the loss by death of Mr. Algernon Sydney, who had done good service for several years as a member of the Council and the Executive Committee, and in many other ways.

I have now only the pleasant task of thanking the members of the Council, members of Committees, visitors, and honorary officers for all their work on behalf of the Fund. I think you would wish me specially to mention Lord Somerleyton, who has been Honorary Secretary of the Fund from its inception, and to congratulate him on the honour recently conferred upon him by His Majesty. (Hear, hear.)

I now beg to move the adoption of the Reports of the Distribution and Convalescent Homes Committees.

The Duke of Norfolk having seconded the motion, the adoption of the Reports was then put and carried unanimously.

On the motion of Lord Farquhar, seconded by Sir Walter Lawrence, it was resolved that the cheques for the grants should be posted on Wednesday, December 20, except in the case of grants for the reservation of beds in consumption sanatoria during 1917, which should be posted on January 1.

### Arrangements for 1917.

Lord Iveagh then said: My Lords and Gentlemen,—I have now to announce the appointments which the Governors propose to make to the Council and Committees for 1917, and you will then be asked to consider the delegation resolutions, which, if approved by you here to-day, will only require to be passed by the formal meeting in January. They are exactly the same as the resolutions already in operation for 1916.

Lord Somerleyton (Hon. Secretary) then read the list of the Council and Committees, including the following new appointments: To the General Council, Mr. W. J. H. Whittall; to the Executive Committee, Sir Edward Hope, K.C.B.

On the motion of Lord Burnham, seconded by Sir Vezey Strong, a resolution was approved delegating powers to the Finance Committee, Distribution Committee, and Convalescent Homes Committee.

On the motion of Sir Henry Burdett, seconded by Sir William Bennett, a resolution was passed delegating further powers to the Finance Committee.

On the motion of Lord Mersey, seconded by Sir Owen Philipps, a resolution was approved delegating to the

### KING EDWARD'S HOSPITAL FUND—(*continued*).

Distribution Committee the power to draw upon the grants set aside for the amalgamation of the Throat Hospitals.

On the motion of Lord Bessborough, seconded by Sir John Tweedy, a resolution was approved delegating powers to the Executive Committee.

### Vote of Thanks to the Chairman.

The Governor of the Bank of England (Lord Cunliffe), in proposing a vote of thanks to Lord Iveagh for presiding, said that in view of his many duties as Governor of the Bank, and the extra work that had fallen on his shoulders during the last two years, he hoped to be excused for not having attended the meetings of the Fund more often.

The resolution was seconded by the President of the Royal College of Physicians (Dr. Frederick Taylor), and carried unanimously.

Lord Iveagh, in acknowledging the vote of thanks, said:—

My Lords and Gentlemen,—I am very grateful for the vote of thanks passed by you. When we met in this room a year ago I have no doubt that in the hearts of many of us there was a hope that our next meeting might be held under more normal conditions, but we meet again while a terrible and ruthless war still rages across Europe, and our year's work has been carried out under the inseparable difficulties brought about by such conditions. Nevertheless we should, I think, be satisfied with the measure of success which has been achieved for the year, success only brought about by the unselfish and self-sacrificing work of those upon whom the bulk of the good work falls. Ours is essentially a work of peace, but I fear many anxious months must pass before peace and its blessings again visit our land. Peace will lessen some of the difficulties under which we are working, but I foresee as one of the results of the hardships and privations of our men in the field, hardships and privations borne so nobly and uncomplainingly, a heavier load even than before will be thrown upon the resources of the hospitals, when these gallant men return to civil life. For in many of them, fighting at the various seats of war, will be sown the seeds of future ills, and it behoves us to continue untiring in our efforts to increase, if possible, the resources of the Fund, instituted through the far-seeing sagacity of His late Majesty King Edward, and in which His Majesty the King continues to take such a deep and encouraging interest.

